# Good Cooking

**Published:** 1867-12

**Authors:** 


GOOD COOKING, and of the ill effects which poor cooking had on the system, he said "the brain depended on the stomach, and if the latter gets out of order the former always follows it. "The seat of reason is dependent upon the seat of digestion." We cannot be sick if our stomach is all right. An intelligent physician always finds out the state of the stomach first, when he visits his patient. What is required to keep the stomach in order, is not much, or costly food, but that which is suitable and proper. A cook, is to a family what an engineer is to an engine. A physician is only needed when the system is out of order, a good engineer knows all about the construction of his engine, and is able to repair it when out of order. A cook ought to have a knowledge of anatomy. Instead of using nostrums, we should use proper food, which is cheaper and easier to take. No chemist is able to imitate nature. In warm climates the fibers of the stomach are softened and extended by heat. Therefore it is necessary to use spices. It is a well-known fact that the Mexicans use immense quantities of red pepper; in fact it is one of the chief articles of diet with them. Their pepper fields are to them what our cornfields are to us. When the crop is harvested it is stored in large sheds. We must not mistake people who are bloated for those who are plump and healthy. Catholic sisters when 40 years of age, look alike because they all live on the same kind of food. The poor whites of the South who live on corn-bread, pork, coffee and whisky, are coarse and brutal. Their eyes have a dull, stupid look. Among all nations the people are refined in proportion to the simplicity and excellence of their food. A cook who is able to read has an advantage over the one who cannot. One who is careful need not buy lard; and if she economizes she will be able to save enough of fat from scraps to make all the soap which is needed in the household. Punctuality is indispensable in a cook, for if her work is not done by a certain hour, her labor is lost and the cooking spoiled. A kitchen should be clean, well ventilated, and dry. The utensils must be of the best quality. There should be a good larder and an ice - box provided. The best housewife can do but little if she have poor materials to do with. The Professor then gave instructions for using the various utensils, and stated what kind were best adapted for general use. Afterward he gave, in plain, simple language, directions for selecting meats, fish and vegetables. In the course of the lecture he said that it is a mistake to drink tea or coffee for breakfast, as tea acts on the nervous systems, and coffee on the blood. Cold water is far better than either. Women who always have been accustomed to drink tea for breakfast, upon leaving off this habit, will feel weak, and hardly able to stand at first, but after a few days they will get over it, and their legs will be stronger than ever. Some people think soup is a kind of slop, containing but little nutriment. On the contrary, the substance of ten pounds of meat can be put in a bowl of soup. Coffee or broth should never be allowed to boil. In coffee the aroma escapes with the steam, and with broth the essence of the meat escapes. A few turkey or beef bones boiled will make enough soup to last a day or two. The crawfish, which are so common in this country, are excellent food, and they are much better here than those found in the old country. Snails are highly esteemed in Germany, France, and Italy. Great quantities of them are found in the vineyards. As they only go an inch or so deep in the ground, they freeze to death during the winter in this country. Sorrel should be freely used in the spring, for it will save many doctor's bills. In making sauces, water should be avoided, and broth used in its stead.

Those who were present seemed highly pleased with the simplicity and clearness of the language used in the lecture. The speaker, in his explanations, makes use of the different kinds of instruments appertaining to cooking. He shows plainly that there is even a right way to pare and cut potatoes, turnips, or carrots. When common sense is introduced with the French art, then people will be inclined to listen to the lectures on cookery.

